# Bilateral Naviculocuneiform Coalition in a Pediatric Patient: A Common Pathology in a Rare Location

**DOI:** 10.7759/cureus.91296

**Published:** 2025-08-30

**Authors:** Tanisha Shankar, Zeeraq Rana, Bryon Thomson

**Affiliations:** 1 Department of Radiology, Western University of Health Sciences, Pomona, USA; 2 Department of Radiology, Riverside University Health System Medical Center, Moreno Valley, USA

**Keywords:** midfoot pain, naviculocuneiform, pediatric foot pain, synchondrosis, tarsal coalition

## Abstract

Tarsal coalition is a condition characterized by abnormal fusion of two or more bones in the hindfoot or midfoot, typically presenting in adolescence with pain, stiffness, or recurrent sprains. While the most common types are calcaneonavicular and talocalcaneal coalitions, naviculocuneiform coalitions are exceedingly rare and often underdiagnosed due to subtle or absent radiographic findings. We report the case of an 11-year-old previously healthy female who presented with left midfoot pain following minor trauma. Physical examination revealed focal tenderness at the medial and dorsal aspects of the midfoot, with preserved range of motion. Initial radiographs showed no acute fractures or dislocation, but marginal spurring at the medial naviculocuneiform joint, an atypical finding in pediatric patients, was noted. Given these imaging findings, an MRI of the foot was obtained and demonstrated cortical irregularity with surrounding edema at the naviculocuneiform joint without marrow continuity, consistent with a non-osseous (likely cartilaginous or fibrous) coalition. Retrospective evaluation of prior contralateral foot radiographs revealed similar findings, supporting a diagnosis of bilateral naviculocuneiform coalition. The patient was referred to podiatry and is currently undergoing conservative management with activity modification and immobilization. Naviculocuneiform coalition is an exceptionally rare variant of tarsal coalition, with minimal documentation in the literature. Epidemiologic studies suggest a much lower incidence compared to more common coalition types, and some populations may exhibit geographic or ethnic predispositions. The presentation is often non-specific, and non-osseous coalitions in particular may evade detection on plain radiographs. MRI plays a crucial role in identifying such coalitions when clinical suspicion is high and initial imaging is inconclusive. Recognition of rare coalition types is important in the differential diagnosis of pediatric midfoot pain to prevent misdiagnosis and guide appropriate management. First-line treatment remains conservative, reserving surgical intervention for refractory or functionally limiting cases. This case highlights a rare presentation of bilateral naviculocuneiform coalition in a pediatric patient, precipitated by minor trauma. It underscores the importance of advanced imaging in evaluating unexplained midfoot pain in young patients and expands the growing body of literature on rare tarsal coalitions. Clinical awareness of such atypical presentations is essential for timely diagnosis and management.

## Introduction

Tarsal coalition is a condition in which two or more bones of the midfoot or hindfoot are fused, resulting in a variety of clinical presentations, ranging from asymptomatic to severely limited foot mobility and pain [1,2]. The documented incidence of tarsal coalition is 1%; however, the true incidence is suspected to be higher due to the number of asymptomatic cases, as evidenced by a cadaveric study [3]. Tarsal coalitions have historically been classified based on their etiology, tissue type, and anatomic location [1,2]. The tissue types include osseous (synostosis), cartilaginous (synchondrosis), or fibrous (syndesmosis). Acquired coalitions can be seen in the adult population and can result from surgery, neoplasm, arthritis, or trauma. The term "tarsal coalition" typically refers to those with a congenital etiology, which are far more common and are thought to be inherited in an autosomal dominant pattern, resulting from the failure of embryonic mesenchyme segmentation [1,2].

Symptoms of congenital tarsal coalitions typically occur in the second decade of life, after the coalitions have progressively begun to ossify, resulting in pain due to compensation for altered joint kinematics. Other aspects of presentation include valgus deformity and subtalar stiffness [2]. It is thought that coalitions that were previously dormant become symptomatic after an episode of trauma, and a history of frequent sprains is a telltale sign. The most common anatomic types of tarsal coalition are talocalcaneal and calcaneonavicular, and the incidence of bilateral tarsal coalitions is estimated to be 50-60% of cases [1]. Coalitions between other tarsal bones, including talonavicular, calcaneocuboid, and naviculocuneoform, have been documented but are far less common [4-9]. Naviculocuneiform coalition is exceptionally rare [4-11].

## Case presentation

**Figure 1:**
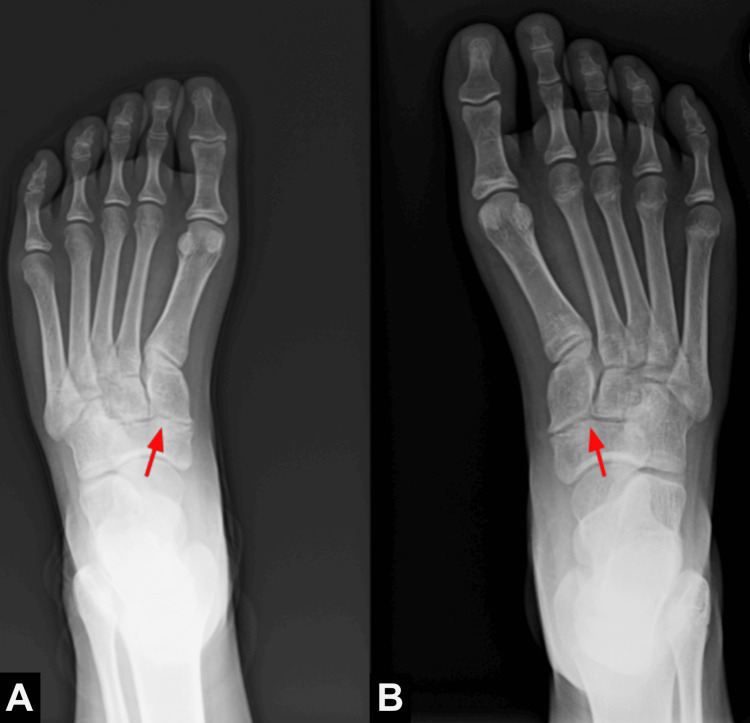
Frontal radiographs of the (A) left and (B) right feet demonstrating bilateral spurring vs. osteophytosis at the medial margins of the naviculocuneiform joints.

An 11-year-old previously healthy female presented to urgent care with left-sided foot pain worse with weight-bearing after sustaining a kick to the dorsum of the foot. Physical exam findings were significant for point tenderness at the medial and dorsal midfoot with a normal range of motion. At the time of initial presentation, the leading concern was for a Lisfranc injury, and urgent left foot radiographs were ordered. Radiographs demonstrated no signs of acute fracture or Lisfranc injury, although marginal spurring versus osteophytosis at the medial naviculocuneiform joint was seen (Figure 1).

**Figure 2:**
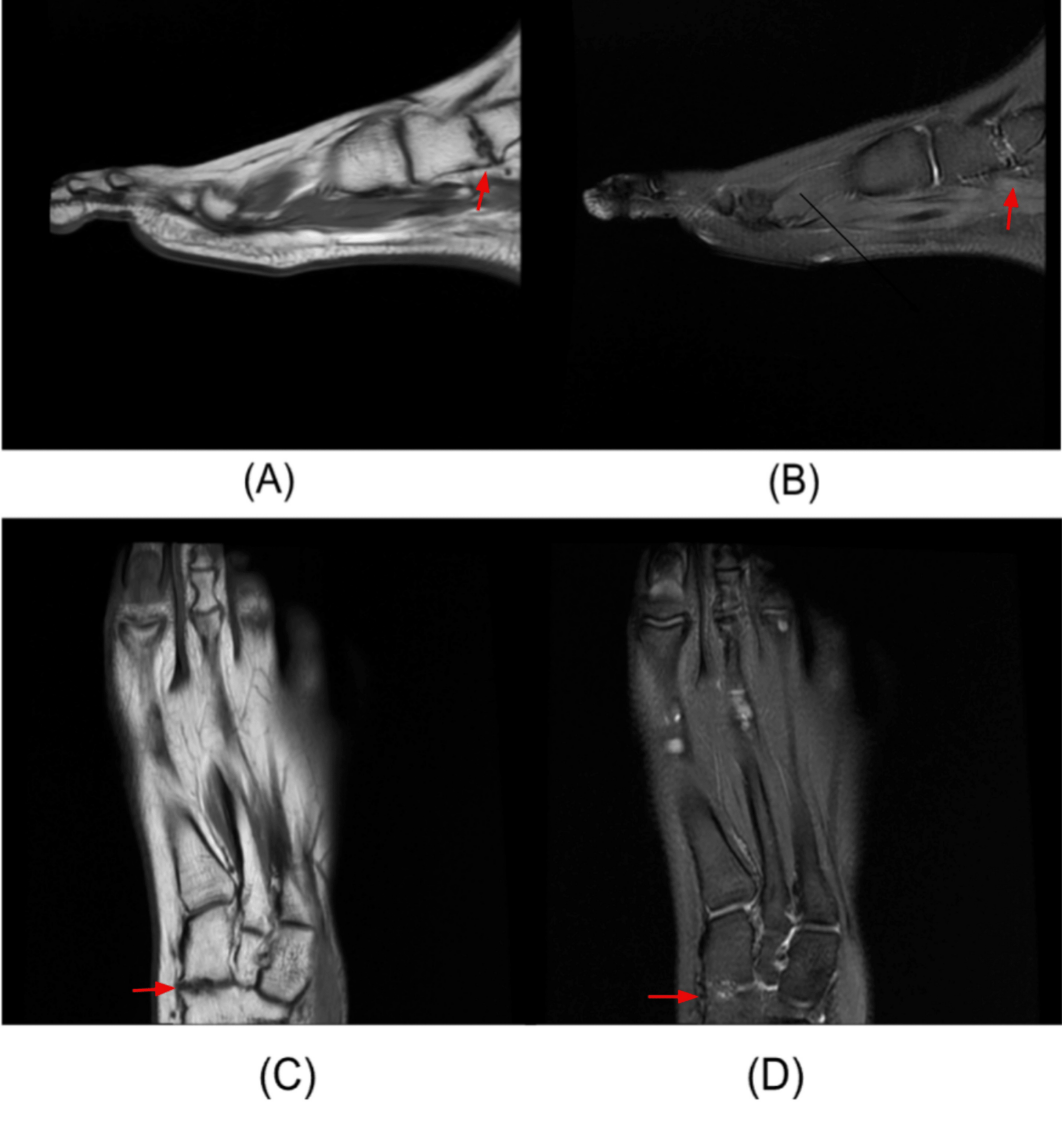
Sagittal T1 (A) and T2 (B) weighted slices and axial T1 (C) and T2 (D) weighted slices of the left foot without contrast, demonstrating a focal cortical irregularity and surrounding edema at the medial margin of the naviculocuneiform joint, without continuity of marrow signal, consistent with a non-osseous coalition.

This finding, common in chronic conditions associated with aging, such as osteoarthritis, was unusual given the patient's young age. Thus, non-emergent follow-up with an MRI was recommended, and the patient was discharged from the urgent care facility with a podiatry referral. MRI of the left foot without contrast demonstrated a focus of cortical irregularity and mild edema at the medial margin of the naviculocuneiform joint, without continuity of marrow signal (Figure 2). Findings were consistent with a non-osseous, cartilaginous coalition. Retrospective analysis of previous contralateral foot radiographs showed similar findings at the medial naviculocuneiform joint, supporting the diagnosis. The patient was seen in the podiatry clinic and is currently undergoing a trial of nonoperative management, including immobilization and limitation of physical activity.

## Discussion

Naviculocuneiform coalition is an exceptionally rare form of tarsal coalition, with limited documentation in the literature. While the overall incidence of tarsal coalition is estimated to be approximately 1%, this figure is likely underestimated due to the high prevalence of asymptomatic cases and missed diagnoses on imaging [1]. The most frequently reported coalitions are calcaneonavicular and talocalcaneal, which together account for over 90% of identified cases [4]. Coalitions involving the talonavicular, calcaneocuboid, and naviculocuneiform joints are significantly less common [4]. In their cadaveric study of 225 feet, Stormont and Peterson did not identify any naviculocuneiform coalitions, highlighting the rarity of this entity [4]. A more recent population-based study from Olmsted County, Minnesota, identified only one case of naviculocuneiform coalition among 79 symptomatic pediatric tarsal coalitions, suggesting an annual incidence of approximately 0.4 per 100,000 children [10]. Furthermore, Burnett and Case, in a study of 1,634 skeletons, found naviculocuneiform coalitions in just 1% of South African specimens, with no cases in European-American or Danish samples, indicating significant geographic and ethnic variation in prevalence [11].

The clinical presentation of tarsal coalition is highly variable and often depends on the patient's age and activity level, the type of coalition, and whether the coalition is osseous or non-osseous. Many patients are asymptomatic until adolescence or early adulthood, when the maturing skeleton, increased physical activity, or trauma may precipitate symptoms. In the present case, an 11-year-old female presented with midfoot pain following direct trauma. Initial radiographs were unremarkable for acute fracture but revealed unusual findings in a pediatric patient that raised concern for an underlying congenital or developmental pathology. MRI played a pivotal role in diagnosis, demonstrating findings consistent with a non-osseous (likely cartilaginous or fibrous) coalition, which may not always be visible on plain films. This highlights the utility of advanced imaging in cases where radiographs are inconclusive, particularly for non-osseous coalitions. Retrospective evaluation of prior contralateral foot radiographs in this case revealed similar changes, suggesting a bilateral process. While the general incidence of bilateral tarsal coalitions is approximately 50%, specific data on bilateral naviculocuneiform coalitions are scarce; however, they have been described in the literature [1,12-14].

The differential diagnosis for midfoot pain in children is broad and includes stress fracture, osteochondrosis, midfoot sprain, accessory bones, and inflammatory arthropathies [10]. Recognition of tarsal coalition, particularly rare types such as naviculocuneiform, is crucial for guiding an appropriate workup and avoiding misdiagnosis.

Treatment of naviculocuneiform coalition depends on the severity of symptoms and the patient's functional status. Conservative management, including rest, NSAIDs (nonsteroidal anti-inflammatory drugs), immobilization, and a custom orthotic, is the first-line approach for most symptomatic cases. Surgical intervention may be indicated in refractory cases or where the coalition significantly impairs function. Options include coalition resection or arthrodesis, depending on the coalition's size, type, and degree of joint degeneration [15]. In this case, conservative management was appropriate, given the likely acute-on-chronic nature of the symptoms and the absence of advanced joint degeneration.

## Conclusions

Naviculocuneiform coalition is a rare form of tarsal coalition that can present subtly and elude diagnosis, particularly in pediatric patients. This case illustrates how non-specific midfoot pain following minor trauma may unmask an underlying congenital anomaly, emphasizing the importance of maintaining a broad differential diagnosis. Advanced imaging modalities, such as MRI, aid in the diagnosis of non-osseous coalitions when radiographic findings are inconclusive. Early recognition is crucial for initiating appropriate conservative treatment and avoiding unnecessary interventions or prolonged symptoms. By contributing to the limited existing literature, this case reinforces the need for clinical awareness of rare coalition types in children presenting with midfoot pain.
